# Surgical Site Infections After Primary Total Knee Arthroplasty: A Retrospective Cohort Study and Critical Assessment of the French ISO-ORTHO Surveillance Indicator

**DOI:** 10.3390/jcm15114047

**Published:** 2026-05-23

**Authors:** Alessander D’Ascoli, Jean-Luc Raynier, Johan Courjon, Yasmina Berrouane, Pascal Boileau, Christophe Trojani

**Affiliations:** 1 Centre National de la Recherche Scientifique (CNRS), Institut National de la Santé et de la Recherche Médicale (INSERM), UMR 7052, U1271, Université Paris Cité, B3OA, 75010 Paris, France; 2 Service de Chirurgie Orthopédique, AP-HP, Hôpital Cochin, 75014 Paris, France; 3 Instutut de Chirurgie Réparatrice Locomoteur et Sports de l’Institut Monégasque de Médecine du Sport (ICR IM2S), 11 Avenue d’Ostende, 98000 Monaco, Monaco; 4 Service de Maladies Infectieuses et Tropicales, Centre Hospitalo-Universitaire de Nice, Hôpital l’Archet 1, 151 Route de Saint Antoine de Ginestière, 06202 Nice, France; 5 Service d’Hygiène Hospitalière, Centre Hospitalo-Universitaire de Nice, Hôpital Cimiez, 4 Avenue Reine Victoria, 06003 Nice, France; 6 Institut de Chirurgie Réparatrice Locomoteur et Sports (ICR Nice), 9 Avenue Durante, 06000 Nice, France

**Keywords:** infection, prosthetic joint infection (PJI), total knee arthroplasty (TKA), healthcare cost, quality of care, indicator

## Abstract

**Background/Objectives:** Prosthetic joint infection following total knee arthroplasty remains a significant public health challenge. Automated surveillance systems are increasingly used for national monitoring of surgical site infections after arthroplasty. This study assessed the performance of the French ISO-ORTHO automated surveillance indicator after primary total knee arthroplasty by comparing automated surveillance data with exhaustive clinical follow-up. It also reported the incidence of surgical site infections during the initial years of activity of a tertiary care university hospital. **Methods:** A retrospective cohort analysis of primary total knee arthroplasties performed between January 2016 and December 2018 was conducted using exhaustive clinical chart review and the French ISO-ORTHO automated surveillance system. Prosthetic joint infections were diagnosed according to the 2018 International Consensus Meeting criteria. The local ISO-ORTHO results were compared with the national ISO-ORTHO rates. **Results:** Clinical chart review identified 1138 primary total knee arthroplasties and five prosthetic joint infections. Prosthetic joint infection incidence was 0.44% with a mean follow-up of 40.5 months. ISO-ORTHO was not yet implemented in 2016. Between 2017 and 2018, ISO-ORTHO identified 519 procedures and one prosthetic joint infection, compared with 807 procedures and three infections identified by clinical review. **Conclusions:** The French ISO-ORTHO surveillance indicator aided local and national monitoring of surgical site infections after total knee arthroplasty, but discrepancies with clinical chart review highlighted important limitations of automated monitoring and the importance of prolonged clinical follow-up. Future surveillance strategies could integrate these complementary approaches to improve prosthetic joint infection detection.

## 1. Introduction

The number of total knee arthroplasties increased by one-third between 2008 and 2013, with projections indicating continued growth through 2030, particularly in the United States [1]. Joint arthroplasty is classified as clean surgery (Altemeier I) [2], where minimizing infection rates is critical [3].

Surgical site infection (SSI), although rare after primary total knee arthroplasty (TKA) (0.5–2%) [4], is associated with poorer functional outcomes after revision surgery [5], higher mortality [6], and costs up to three times greater than uncomplicated procedures [7,8]. SSI is often considered the primary cause of TKA revision, surpassing aseptic loosening [9,10]. The reported risk factors include diabetes; smoking; obesity; American Society of Anesthesiologists (ASA) score ≥ 3 reflecting greater systemic comorbidity burden; prolonged surgery; and National Nosocomial Infection Surveillance (NNIS) score ≥ 1, which combines ASA score, wound classification, and operative duration [11,12,13,14].

Risk management strategies, including optimization of patient-related factors, operating room standards, and antibiotic prophylaxis, have contributed to reducing SSI rates [15,16]. Reliable surveillance strategies are therefore essential in monitoring SSI rates and identifying atypical infection patterns across institutions.

Registry-based surveillance systems are increasingly used to monitor SSI rates and identify atypical infection patterns across healthcare institutions [17]. Automated surveillance tools may facilitate both local and large-scale SSI monitoring while enabling standardized inter-institutional comparisons [18].

The French ISO-ORTHO indicator is an automated national surveillance tool based on the French national hospital discharge database for medicine, surgery and obstetrics (Programme de Médicalisation des Systèmes d’Information Médecine-Chirurgie-Obstétrique (PMSI-MCO)). It monitors surgical site infections occurring within three months after primary total hip or knee arthroplasty in public hospitals and enables standardized comparison of institutional infection rates at a national level. The indicator provides both crude infection rates and standardized infection ratios. The standardized ISO-ORTHO ratio corresponds to the number of observed SSIs divided by the number of expected SSIs, allowing comparison of institutional infection rates at a national level and classification of centers as atypically high, low or within the expected range according to standard deviation thresholds [19]. However, discrepancies between automated surveillance systems and prolonged clinical follow-up may still occur, particularly when relying on administrative databases and short-term surveillance.

This study evaluated the ISO-ORTHO indicator through comparison with prolonged clinical follow-up after primary TKA. Secondary objectives were to report local SSI incidence and compare institutional results with national reference data during the initial years of activity of a tertiary care university hospital.

## 2. Material and Methods

### 2.1. Study Design and Population

This retrospective single-center study included all the primary TKAs performed from 1 January 2016 to 31 December 2018, during the initial years of activity of a tertiary care university hospital (Hospital Pasteur 2, Nice, France), opened in June 2015. This retrospective observational study used anonymized routine care data and complied with the French MR-004 reference methodology for health data research. According to the local Ethics Committee, formal ethical approval was not required.

**Figure 1:**
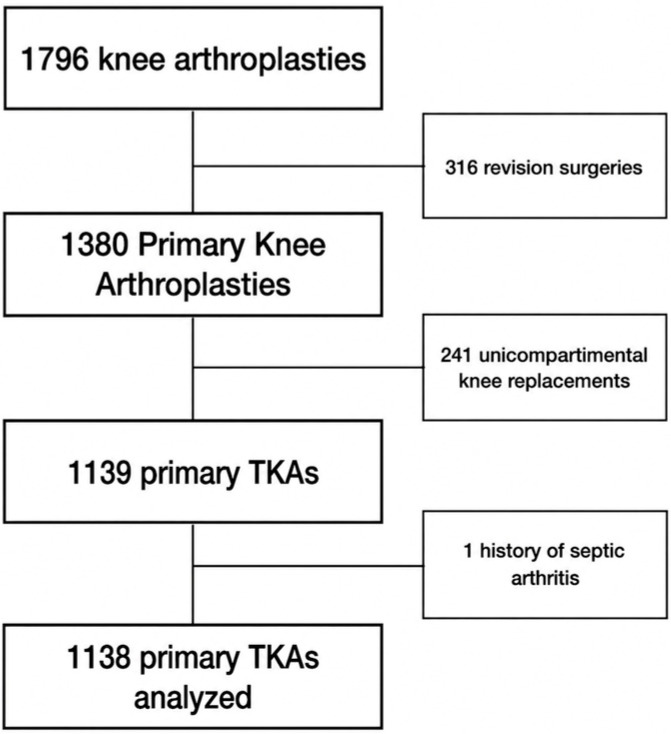
Flow-chart.

During the study period, 1380 primary knee arthroplasties were performed. A total of 241 unicompartmental knee arthroplasties were excluded, yielding 1139 primary total knee arthroplasties (TKAs). The exclusion criteria included prior septic arthritis (*n* = 1) and TKAs performed for distal femoral or tibial plateau fractures (Figure 1).

### 2.2. Surgical Procedures and Follow-Up

Patients were hospitalized the day before surgery according to the institutional protocol. Pre-anesthetic and surgical assessments were performed before surgery. Povidone–iodine decontamination was performed before surgery and repeated in the operating room under sterile conditions. All the orthopedic operating rooms had vertical laminar airflow ventilation [20], and the procedures were performed under a pneumatic tourniquet. Two posterior-stabilized cemented implants were used, with systematic patellar resurfacing. NexGen LPS^®^ (Zimmer, Warsaw, IN, USA) was used in 893 cases and Anatomic^®^ (Amplitude, Valence, France) in 245 cases. Follow-up visits were scheduled at 45 days, 90 days, 6 months, 1 year, and 2 years postoperatively, with additional assessments as needed.

### 2.3. Definition and Management of Prosthetic Joint Infections

Suspected periprosthetic joint infections (PJIs) were discussed through a multidisciplinary approach. Diagnosis followed French National Authority for Health (HAS) and Infectious Diseases Society of America (IDSA) guidelines and considered the International Consensus Meeting criteria [21]. All the knee osteoarticular bacteriological results (*n* = 4419) from 1 January 2016 to 30 April 2025 were reviewed to identify potentially missed PJIs. This approach aimed to maximize PJI detection through prolonged clinical and microbiological surveillance.

The PJI treatment included debridement, antibiotics, and implant retention (DAIR) for acute infections (<1 month) and one- or two-stage revision arthroplasty for chronic infections (>1 month). Acute infections were diagnosed based on clinical presentation, biological findings, and imaging when required, whereas joint aspiration was systematically performed in suspected chronic infections. In cases of negative aspiration despite persistent suspicion of infection, scintigraphy was systematically performed. Positive aspiration or scintigraphy results indicated infection. Broad-spectrum intravenous dual antibiotic therapy (PIPERACILLIN + TAZOBACTAM, DAPTOMYCIN) was initiated intraoperatively after collection of deep bacteriological samples. Antibiotic therapy was subsequently adapted to culture results for a total duration of 6 weeks. Revisions for mechanical loosening systematically included 3–5 intraoperative bacteriological samples. More than one positive sample indicated PJI, and antibiotic therapy was initiated in the orthopedic unit. Follow-up by orthopedic surgeons and infectious disease specialists continued for a minimum of 2 years.

### 2.4. ISO-ORTHO Methodology

Institutional and national ISO-ORTHO data were collected for comparison. The ISO-ORTHO analysis was based on PMSI-MCO hospital discharge data, a French nationwide administrative database in which all hospital stays, diagnoses, and procedures are prospectively coded for medical, surgical, and obstetrical activity reporting. Using these coded data, ISO-ORTHO first identifies total hip and knee arthroplasties performed in French public hospitals and subsequently detects surgical site infections associated with these procedures within 3 months after surgery. The Standardized ISO-ORTHO Ratio (RS-ISO-ORTHO) is applied to non-fracture TKAs and corresponds to the ratio between observed and expected SSIs derived from national PMSI-MCO data. Institutions were classified as atypically high, low or within the expected range according to standard deviation thresholds (2 standard deviations thresholds in 2017 and 3 standard deviations thresholds from 2018 onward). ISO-ORTHO data were analyzed for patients operated on in 2017 and 2018, since the indicator was not active in 2016.

### 2.5. Statistical Analysis

PJI incidence rates were calculated annually and for the overall study period. Comparisons between groups were performed using Student’s *t*-test and Fisher’s exact test. Statistical analyses were performed using the SPSS software version 27.0 (IBM Corp., Armonk, NY, USA). A *p*-value < 0.05 was considered statistically significant.

## 3. Results

### 3.1. Clinical Results

The incidence of SSIs after primary TKAs during the study period was 0.44% (5/1138) with a mean follow-up of 40.45 months (range, 25–74 months). This included three acute and two chronic infections. A review of all 4419 knee bacteriological samples obtained in our center from 2016 to 2024 found no additional SSIs. Three revision procedures performed for mechanical loosening yielded one positive culture for *S. epidermidis* in one of five samples, and were considered contamination. No antibiotics were administered, and revision surgery was successful, with favorable outcomes at 2 years and no evidence of PJI. Population characteristics of the overall cohort and PJI cases are presented in Table 1. The patients with PJI had significantly higher ASA and NNIS scores. No other significant differences were observed, likely because of the small number of PJI cases.

Thirty-two patients did not attend their two-year clinical follow-up visit and were assessed by phone consultation. Twenty-six had satisfactory outcomes, while six underwent revision surgery elsewhere for aseptic loosening. All had negative labeled leukocyte scintigraphy, and one also had negative preoperative joint aspiration.

Seventeen revision procedures performed for suspected mechanical loosening at our center showed negative preoperative aspiration, aseptic loosening on bone scintigraphy, and negative intraoperative cultures.

### 3.2. ISO-ORTHO Results

In 2016, 331 primary TKAs were performed, but the ISO-ORTHO indicator was not yet active. One patient required DAIR for acute infection at day 20, and another underwent single-stage revision for chronic *S. aureus* infection at four months.

In 2017, 387 primary TKAs were performed, and one chronic SSI was identified during record review. The ISO-ORTHO tool reported 248 procedures with no SSIs at three months.

In 2018, 420 primary TKAs were performed with two identified SSIs, whereas ISO-ORTHO reported only 271 procedures and one SSI diagnosed within 3 months (Table 2).

### 3.3. Comparative Analysis of ISO-ORTHO Detection

Overall, our review identified 1138 primary TKAs and five PJIs. Restricting the analysis to procedures performed in 2017 and 2018, we found 807 TKAs and three PJIs, whereas the ISO-ORTHO indicator identified only 519 procedures and one PJI during the same period. One chronic infection fell outside the 3-month surveillance period, while one acute infection was not detected by the automated indicator. In addition, 288 procedures were not identified by ISO-ORTHO.

In 2017, our institution was classified as “atypical low”, with an SSI rate more than two standard deviations below the national average. Only 13 of 731 French institutions (1.8%) were classified as atypical low that year. The next year, our institution’s score remained below two standard deviations, but the threshold for atypical classification changed from two to three standard deviations, leading to classification within the standard range. A similar effect was observed for institutions previously classified as atypically high. That year, only 1.3% of healthcare facilities were classified as atypically high or low. No detailed institutional characteristics were available for these facilities.

### 3.4. Characteristics and Outcomes of PJI Cases

One acute infection caused by *Streptococcus pyogenes* led to sepsis-related death in a high-risk patient, while another polymicrobial infection (*Methicillin-Resistant Staphylococcus aureus, Enterobacter cloacae*) required multiple surgeries. All the other patients had favorable outcomes after a single surgical procedure. The clinical and microbiological characteristics of infected patients are presented in Table 3.

## 4. Discussion

In this study, the SSI rate after primary TKA was 0.44% at a minimum follow-up of 2 years. The reported PJI rates after TKA vary substantially across studies, ranging from 0.39% to 3.9% depending on study design, surveillance methods and duration of follow-up [22]. For example, Grammatico et al. [23] reported a 1.31% PJI rate at 1 year, whereas Kurtz et al. [16] found 1.55% at 2 years in the Medicare population. The reported rates of PJI after total hip arthroplasty also show substantial variability across studies and surveillance systems, ranging from approximately 0.5% to 2% depending on follow-up duration and case definition [17,24]. To improve standardization of surveillance, automated systems based on administrative and clinical databases have been developed for large-scale monitoring of SSIs. However, the accuracy of these systems remains highly dependent on coding strategies and the quality of available clinical information [25]. Kandel et al. [26] demonstrated in a series of nearly 28,000 hip and knee arthroplasties that combining diagnosis and procedural codes improved automated PJI detection.

The ISO-ORTHO indicator was developed in France to provide standardized national surveillance of surgical site infections after hip and knee arthroplasty based on administrative hospital discharge data (PMSI-MCO) with a fixed 3-month postoperative follow-up window. In our study, applying the same 3-month timeframe, the ISO-ORTHO tool identified fewer procedures and fewer prosthetic joint infections than the clinical review. These discrepancies were mainly related to errors and incompleteness in administrative coding, which led both to missed PJI and to incomplete identification of TKA procedures. These findings highlight the limitations of relying solely on administrative coding-based surveillance systems.

The 3-month surveillance window of the ISO-ORTHO indicator also represents a major limitation, as previous studies have shown that a substantial proportion of PJIs are diagnosed beyond the early postoperative period [27].

Despite its limitations, ISO-ORTHO remains the only nationwide standardized tool available in France for surveillance of PJI after hip and knee arthroplasty and therefore represents an essential instrument for institutional and national monitoring. Its improvement is necessary, since SSI incidence is widely considered a marker of quality of care [28,29], and should rely on complementary strategies such as systematic review of medical records in selected cases and a strengthened multidisciplinary approach. These observations led us to the implementation of a double verification process, including postoperative coding review, to improve detection of procedures and PJIs. From 2019, collaboration with the hospital hygiene team ensured tool reliability.

SSI reduction is ethically and economically crucial, with Kurtz et al. [8] noting $74,900 additional costs per patient and Puhto et al. [30] reporting triple the cost versus primary arthroplasty. Although the present study was not designed to identify specific risk factors for PJI, well-established factors described in the literature include comorbidity such as diabetes, obesity, high ASA score, and prolonged surgical site duration [24,31,32]. Specific skin preparation protocols [3] and other less well-characterized perioperative factors may also contribute to variations in PJI risk, although their precise impact remains difficult to quantify.

In this context, the classification of institutions as “atypically high” or “atypically low” within the ISO-ORTHO framework can be used to further study these outlier centers and to better understand patient and system-related determinants.

The ISO-ORTHO standardized ratio is based on a threshold of standard deviations from the national mean, which was secondarily modified from two to three standard deviations, resulting in a reduction in the number of institutions classified as outliers. This change in threshold likely reduces the sensitivity of the indicator and limits overall interpretation, potentially leading to under-detection of hospitals with elevated infection rates.

This study has several limitations, including its retrospective nature. Despite a large cohort, the relatively small number of PJIs limits the statistical analysis. In addition, the single-center setting may limit the generalizability of the findings. The main strength of this study is the use of a complete institutional cohort with systematic follow-up, allowing an extensive search for PJI and postoperative complications, and allowing a direct comparison between clinical diagnosis and administrative surveillance.

## 5. Conclusions

This study demonstrates a discrepancy between clinically confirmed prosthetic joint infections and those identified through the ISO-ORTHO administrative surveillance system, which also missed several total knee arthroplasty procedures. While ISO-ORTHO remains a useful national tool, its performance is limited by coding accuracy and the fixed 3-month postoperative surveillance window. These findings suggest that improving surveillance quality requires not only refinement at the national level but also efforts at the local level, including improved coding practices, systematic clinical review, and multidisciplinary validation.

## Figures and Tables

**Table 1:** Population characteristics.

	Primary TKA Population (*n* = 1138)	PJI Population (*n* = 5)	*p* Value
age (y)	70.27 [21; 94]	66.2 [50; 76]	0.618
sex Ratio (M/F)	0.36 (410/1138)	0.4 (2/5)	1
ASA score	2.01 [1; 4]	2.8 [2; 4]	0.019
ASA ≥ 3 (%)	19.4 (221/1138)	60 (3/5)	0.054
diabetes (%)	11.16 (127/1138)	40 (2/5)	0.101
BMI (kg/m^2^)	28.68 [16.9; 50.8]	32.45 [26.2; 44.4]	0.321
smoker (%)	7.38 (84/1138)	20 (1/5)	0.321
NNIS score	0.34 [0; 2]	1.2 [0; 2]	<0.001
operating time (min)	72.34 [19; 123]	86.62 [44; 120]	0.220

Quantitative variables are presented as mean [range] and qualitative variables as percentages. TKA: total knee arthroplasty; PJI: prosthetic joint infection; ASA: American Society of Anesthesiologists; BMI: Body Mass Index; NNIS: National Nosocomial Infections Surveillance.

**Table 2:** PJI incidence and ISO-ORTHO data.

Surgery Year	2016	2017	2018
Primary TKAs performed	331	387	420
Primary TKAs identified by ISO-ORTHO	/	248	271
Institutional PJI incidence	2/331 (0.6%) acute: 1/331 (0.3%) chronic: 1/331 (0.3%)	1/387 (0.25%) acute: 0/387 chronic: 1/387 (0.25%)	2/420 (0.48%) acute: 2/420 (0.48%) chronic: 0/420
ISO-ORTHO institutional PJI incidence	/	0/248 (0%)	1/271 (0.37%)
ISO-ORTHO national PJI incidence	/	1.05%	0.86%
ISO-ORTHO standardized ratio	/	Atypical low (<−2SD)	Standard (between −2 and −3SD)

TKA: total knee arthroplasty; PJI: prosthetic joint infection; SD: standard deviation.

**Table 3:** Epidemiology of treated PJI.

Infected TKA	Patient A	Patient B	Patient C	Patient D	Patient E
Age (year)	70	68	67	50	76
Sex (male or female)	female	male	female	male	female
BMI (kg/m^2^)	30.5	31.2	26.2	29.8	44.4
ASA score	3	2	3	2	4
Immunodepression	No	No	Yes	No	No
Diabetes	Yes	No	No	No	Yes
Year of surgery	2016	2016	2017	2018	2018
Infection type	Acute	Chronic	Chronic	Acute	Acute
Time before diagnosis (days)	14	118	518	21	29
Isolated pathogens	*S. aureus* *E. cloacae*	*S. aureus*	*C. striatum*	*S. aureus*	*S. pyogenes* *P. stutzeri*
Pathogen phenotype	Methicillin-R	Wild type	Wild type	Wild type	Wild type
Surgical treatment	DAIR +1 stage revision	1-stage revision	2-stage revision	DAIR	DAIR
Status at last follow-up	Healed	Healed	Healed	Healed	Deceased from PJI-related sepsis

TKA: total knee arthroplasty; BMI: Body Mass Index, ASA: American Society of Anesthesiologists; DAIR: debridement, antibiotics and implant retention; PJI: prosthetic joint infection.

## Data Availability

The original contributions presented in this study are included in the article. Further inquiries can be directed to the corresponding author.
